# Genomic evidence that Ornithinicoccus soli Jiang et al. 2020 is a later heterotypic synonym of Segeticoccus rhizosphaerae Lee and Whang 2020

**DOI:** 10.1099/ijsem.0.006503

**Published:** 2024-08-27

**Authors:** Qing Liu, Yu-Hua Xin

**Affiliations:** 1 China General Microbiological Culture Collection Center, Institute of Microbiology, Chinese Academy of Sciences, Beijing 100101, PR China

**Keywords:** heterotypic synonym, *Ornithinicoccus soli*, *Segeticoccus rhizosphaerae*

## Abstract

The genus *Segeticoccus* includes only one species with validly published name, *Segeticoccus rhizosphaerae*. The type strain *Segeticoccus rhizosphaerae* YJ01^T^ was isolated from soil in Korea, while *Ornithinicoccus soli* XNB-1^T^ was isolated from soil in China. Both strains share similar phenotypic and chemotaxonomic characteristics, including predominant menaquinone MK-8(H_4_) and major polar lipids diphosphatidylglycerol and phosphatidylglycerol. Whole genome sequences revealed a DNA G+C content of 70.1 mol% for both strains and 100% similarity in their 16S rRNA gene sequences. Phylogenetic analysis showed they form a distinct cluster separate from other genera. Genomic comparisons showed average nucleotide identity and digital DNA–DNA hybridization values of 99.16 and 94.2%, respectively, indicating they represent a single species. Based on this genomic evidence, *Ornithinicoccus soli* Jiang *et al*. 2020 is proposed to be a later heterotypic synonym of *Segeticoccus rhizosphaerae* Lee and Whang 2020.

The genus *Segeticoccus*, was first described by Lee and Whang [1]. Currently, only one species within *Segeticoccus* is validly published, namely, *Segeticoccus rhizosphaerae* [2]. The type strain *Segeticoccus rhizosphaerae* YJ01^T^ (=KACC 19547^T^=NBRC 113173^T^), was isolated from a spinach farming field soil at Shinan in Korea. It was a Gram-stain-positive, aerobic, non-motile, non-spore-forming cocci, capable of growing at 10–37 °C (optimum, 28–30 °C) and 0–7.5 % (w/v) NaCl (optimum, 1.0% NaCl) [1]. *Ornithinicoccus soli* XNB-1^T^ (=CCTCC AB 2019099^T^=KCTC 49259^T^) was isolated from farmland soil in Taian, Shandong province, PR China [3]. It is a Gram-stain-positive, aerobic, non-motile, coccoid-shaped bacterium, capable of growing at 16–30 °C (optimum, 30 °C) and 0–7% (w/v) NaCl (optimum, 1.0%). Both strains contained MK-8(H_4_) as the predominant menaquinone, and diphosphatidylglycerol and phosphatidylglycerol as the major polar lipids. Both the names *Segeticoccus rhizosphaerae* Lee and Whang 2020 [1] and *Ornithinicoccus soli* Jiang *et al*. 2020 [3] were published in issue 3 of volume 70 in 2020. However, their relationship and taxonomic status need to further clarified.

Whole genome sequences of type strains in the genera *Segeticoccus*, *Ornithinicoccus*, *Ornithinimicrobium*, and related genera within family *Intrasporangiaceae* were obtained from the publicly available NCBI GenBank. Genome quality, genome completeness and contamination were evaluated using the CheckM program version 1.1.3 [4]. The genome sequences were annotated using Prokka v1.13 software [5]. The 16S rRNA gene phylogenetic trees were reconstructed by the neighbour-joining (NJ) [6] and maximum-likelihood (ML) [7] methods using the mega software version 5.2 [8]. The alignment was generated using mafft version 7.520 software [9]. The genetic distances for the NJ analysis were calculated by Kimura’s two parameter model [10]. The ML phylogenetic tree was generated with the best nucleotide substitution model of GTR+G+I [11]. The tree topologies were evaluated by bootstrap values based on 1000 resamplings [12]. The phylogenomic tree was reconstructed usingiq-tree version 2.0.7 [13] with model GTR+F+R5 based on 92 core genes extracted by UBCG program [14]. The digital DNA–DNA hybridization (dDDH) values were calculated using the TYGS as implemented on the DSMZ website [15]. The average nucleotide identity (ANI) values were determined using FastANI version 1.33 [16]. The average amino acid identity (AAI) values were calculated using the CompareM program (https://github.com/dparks1134/CompareM).

The genome information showed that the DNA G+C content of both strains was 70.1 mol% (Table 1). The complete 16S rRNA gene sequences of *Segeticoccus rhizosphaerae* YJ01^T^ (1519 bp) and *Ornithinicoccus* soli XNB-1^T^ (1519 bp) were retrieved from their genomic sequences, revealing 100% similarity, indicating a very close relationship. The 16S rRNA gene phylogenetic tree showed that *Segeticoccus rhizosphaerae* and *Ornithinicoccus soli* formed a distinct cluster, separated from *Ornithinicoccus halotolerans* and *Ornithinicoccus hortensis* (Figs 1 and S1, available in the online Supplementary Material).

**Table 1.** Genomic information of *Segeticoccus rhizosphaerae* YJ01^T^ (GCA_009192725.1) and *Ornithinicoccus soli* XNB-1^T^ (GCA_005222685.1)

Strains	G+C (mol%)	Size (Mb)	Contigs	Genes	Proteins	Completeness (%)	Contamination (%)
*Segeticoccus rhizosphaerae* YJ01^T^	70.1	4.43	759	4683	4287	99.05	0.54
*Ornithinicoccus soli* XNB-1^T^	70.1	4.56	107	4325	4180	100	3.22

**Fig. 1.**
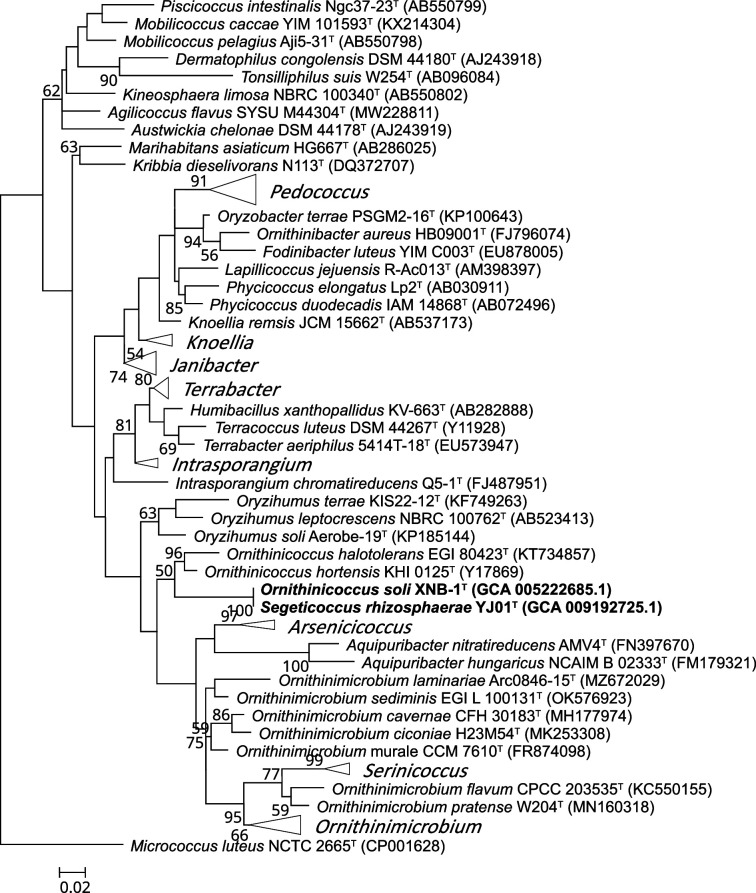
Phylogenetic tree based on the 16S rRNA gene sequence comparisons using the maximum-likelihood method. GenBank accession numbers of the 16S rRNA gene sequences are given in parentheses. Bootstrap values (>50%) based on 1000 replicates are shown at the branch nodes. Bar, 0.02 substitutions per nucleotide positions.

A robust phylogenomic tree showed the very close relationship between *Segeticoccus rhizosphaerae* YJ01^T^ and *Ornithinicoccus soli* XNB-1^T^, indicating they may belong to the same species (Fig. 2). They formed an independent branch, separated from other genera such as *Ornithinicoccus*, *Oryzihumus* and *Ornithinimicrobium*. Therefore, strain XNB-1^T^ should be classified as belonging to the genus *Segeticoccus* rather than *Ornithinicoccus*. The ANI and dDDH values between the two strains were 99.1 and 94.2%, respectively, confirming that they represent a single species. The ANI values between *Segeticoccus rhizosphaerae* YJ01^T^, *Ornithinicoccus soli* XNB-1^T^ and other type strains of genus *Ornithinicoccus* were lower than 78% (Table 2). The AAI values between *Segeticoccus rhizosphaerae* YJ01^T^, *Ornithinicoccus soli* XNB-1^T^ and other members of *Ornithinicoccus* were lower than 63.7%, which were below the AAI threshold for bacterial genus delineation of 65–72% suggested by Konstantinidis and Tiedje [17]. Therefore, combined with the evidence provided by Lee and Whang [1], they should be classified into the genus *Segeticoccus*. Additionally, both strains shared similar phenotypic and chemotaxonomic characteristics, as shown in Table 3.

**Fig. 2.**
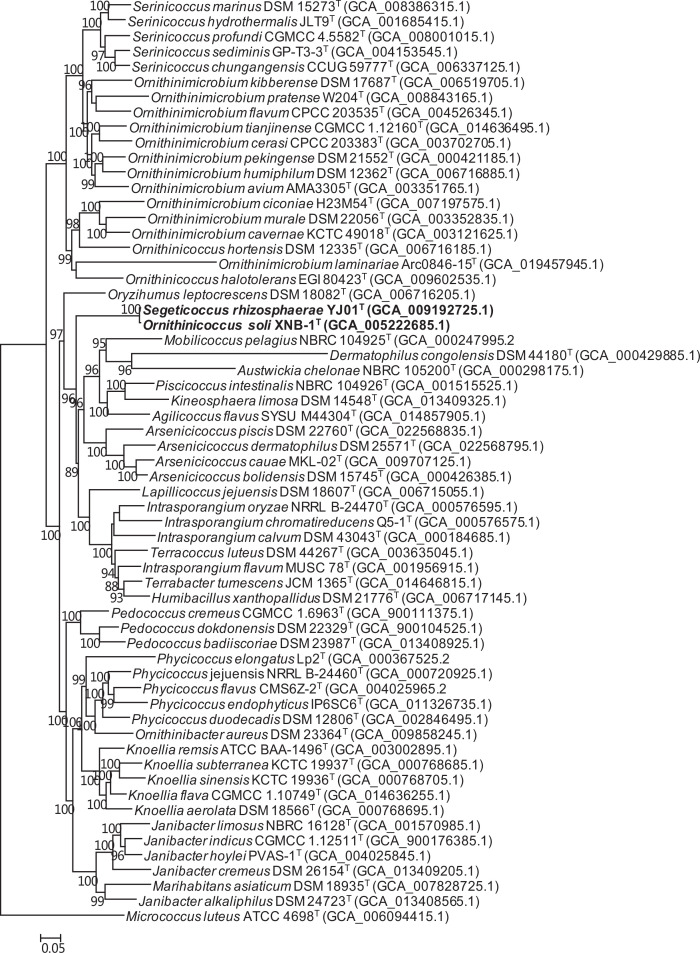
Phylogenomic tree showing the relationship of *Ornithinicoccus soli*, *Segeticoccus rhizosphaerae*, and related species, reconstructed based on concatenated alignment of 92 core genes using the nucleotide substitution model of GTR+F+R5. Bootstrap values (>70%) based on 1000 replicates are shown at branching points. Bar, 0.05 substitutions per nucleotide positions.

**Table 2.** Average nucleotide identity values (%) between *Segeticoccus rhizosphaerae* YJ01^T^ and type strains of the genus *Ornithinicoccus* Strains: 1*, Segeticoccus rhizosphaerae* YJ01^T^; 2, *Ornithinicoccus soli* XNB-1^T^; 3, *Ornithinicoccus halotolerans* EGI 80423^T^; 4, *Ornithinicoccus hortensis* DSM 12335^T^.

Strain	1	2	3
**2**	99.1		
**3**	77.2	77.1	
**4**	77.2	77.2	79.6

**Table 3.** Phenotypic characteristics of *Segeticoccus rhizosphaerae* YJ01^T^ and *Ornithinicoccus soli* XNB-1^T^ Strains: 1*, Segeticoccus rhizosphaerae* YJ01^T^; 2, *Ornithinicoccus soli* XNB-1^T^. +, Positive, –, negative.

Characteristic	1*	2*
Cell morphology	Cocci	Cocci
Colony colour	Yellow	Pale yellow
Optimum temperature for growth (°C)	30	28–30
NaCl range (optimum) for growth (%, w/v)	0–7.5 (1)	0–10 (1)
Motility	+	+
Hydrolysis of:		
Casein	–	–
Gelatin	–	–
Enzymatic activities:		
Alkaline phosphatase	+	+
Esterase lipase (C8)	+	+
Valine arylamidase	+	+
Leucine arylamidase	+	+
Cystine arylamidase	+	+
Trypsin	+	+
Acid phosphatase	+	+
*α*-Glucosidase	+	+
Esterase (C4)	–	+
Naphthol-AS-BI-phosphohydrolase	–	+
*β*-Glucuronidase	–	+
Major polar lipids	DPG, PG, PS, PGL, PAL, PGAL	DPG, PG, PI, PLs, PS
Predominant menaquinone	MK-8(H_4_)	MK-8(H_4_)

+,Positive, –, negative. *, Data from Lee and Whang [1], and Jiang *et al*. [3]. DPG: diphosphatidylglycerol; PG, phosphatidylglycerol; PI: phosphatidylinositol; PGL unknown phosphoglycolipid; PS, phosphatidylserine; PAL, unidentified phosphoaminolipid; PLs, unidentified phospholipids; PGAL, unidentified phosphoglycoaminolipid.

Collectively, based on the genomic evidence, including ANI, dDDH and AAI values, and phylogenetic characteristics, *Ornithinicoccus soli* is proposed to be a later heterotypic synonym of *Segeticoccus rhizosphaerae*.

## supplementary material

10.1099/ijsem.0.006503 Uncited Fig. S1.

## Abbreviations

AAI: average amino acid identity
ANI: average nucleotide identity
dDDH: digital DNA–DNA hybridization
ML: maximum-likelihood
NJ: neighbor-joining
