# Supplementary material for: Genomic evidence that Ornithinicoccus soli Jiang et al. 2020 is a later heterotypic synonym of Segeticoccus rhizosphaerae Lee and Whang 2020
Source: Int J Syst Evol Microbiol. 2024 Aug 27;74(8):006503. doi: 10.1099/ijsem.0.006503 (PMC11350378; doi:10.1099/ijsem.0.006503)
Supplement: Uncited Fig. S1. [file ijsem-74-06503-s001.pdf]

# **Genomic evidence that *Ornithinicoccus soli* Jiang et al. 2020 is a later heterotypic synonym of *Segeticoccus rhizosphaerae* Lee and Whang 2020**

Qing Liu, Yu-Hua Xin*

***Corresponding author:**

Yu-Hua Xin, Email: xinyh@im.ac.cn

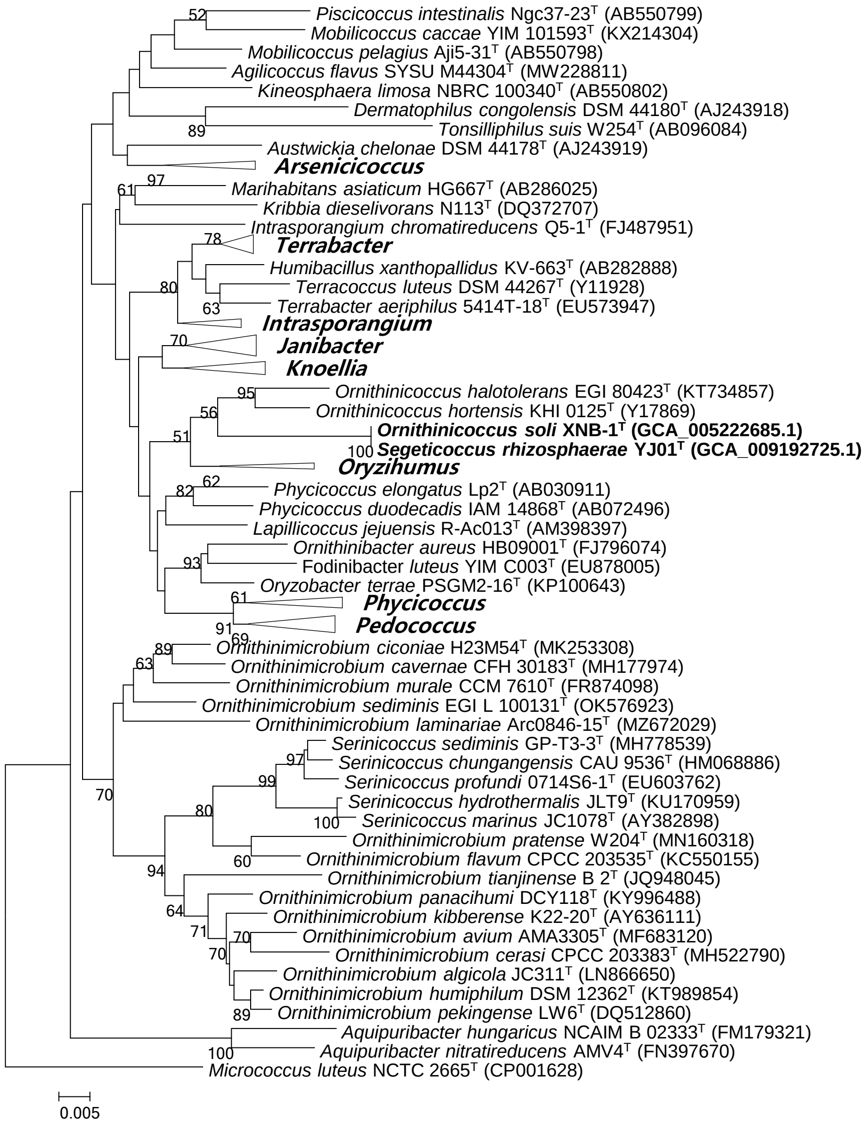


**Fig. S1.** Phylogenetic tree based on the 16S rRNA gene sequence comparisons using the NJ method. GenBank accession numbers of the 16S rRNA gene sequences are given in parentheses. Bootstrap values (>50 %) based on 1,000 replicates are shown at the branch nodes. Bar, 0.005 substitutions per nucleotide positions.
